# Mushroom *β*-Glucan May Immunomodulate the Tumor-Associated Macrophages in the Lewis Lung Carcinoma

**DOI:** 10.1155/2015/604385

**Published:** 2015-06-17

**Authors:** Wan-Jhen Wang, Yu-Sheng Wu, Sherwin Chen, Chi-Feng Liu, Shiu-Nan Chen

**Affiliations:** ^1^College of Life Sciences, National Taiwan University, No. 1, Sec. 4, Roosevelt Road, Da' an District, Taipei City 10617, Taiwan; ^2^Department of Research and Development, Super Beta Glucan Inc., Irvine, CA, USA; ^3^Graduate Institute of Integration of Traditional Chinese Medicine with Western Nursing, National Taipei University of Nursing and Health Sciences, Taipei, Taiwan

## Abstract

The present study showed that oral mushroom beta-glucan treatment significantly increased IFN-*γ* mRNA expression but significantly reduced COX-2 mRNA expression within the lung. For LLC tumor model, oral *Ganoderma lucidum* or *Antrodia camphorata* polysaccharides treatments significantly reduced TGF-*β* production in serum. In addition, IL-12 and IFN-*γ* mRNA expression were significantly increased, but IL-6, IL-10, COX-2, and TGF-*β* mRNA expression were substantially following oral mushroom polysaccharides treatments. The study highlights the efficacious effect of mushroom polysaccharides for ameliorating the immune suppression in the tumor microenvironment. Increased M1 phenotype of tumor-associated macrophages and attenuated M2 phenotype of tumor-associated macrophages could be achieved by ingesting mushroom polysaccharides.

## 1. Introduction

Tumor cells affect the surrounding cellular environment by promoting tumor growth and metastasis via establishment of a tumor microenvironment conducive to tumor development [1–5]. Tumor cells secrete inflammatory cytokines, such as transforming growth factor-*β* (TGF-*β*) and interleukin-10 (IL-10), that stimulate differentiation of regulatory T and Treg cells [6–10], as well as differentiation of tumor-associated macrophages (TAMs) into M2 macrophages, leading to host immune response and subsequent tumor cell evasion of this immune surveillance and attack, in turn enhancing tumor growth and metastasis [1, 11–17]. Various cytokines, chemokines, and growth factors are the primary elements in affecting the host antitumor ability and host evasion of tumor cells [3, 18]. Macrophages are the most important and abundant immune cells and there are primarily two types of macrophages based on function and differentiation: classically activated macrophage (M1 macrophage) and alternatively activated macrophage (M2 macrophage). M1 macrophages are characterized by tumor resistance, while M2 macrophages are characterized by tumor promotion [16, 19]. In mice models, macrophages present CD11b, F4/80, and colony-stimulating factor-1 receptor (CSF-1R), with F4/80 being the surface proteins for M1 and M2 macrophages [11, 20].

TAMs have the major role in the tumor microenvironment to bear immune inhibitory effect [20, 21]. Tumor cells and the surrounding stoma cells secrete cytokines and growth factors that stimulate TAMs and activate the various expressions, functions, receptor regulations, and secretions of chemokines [22, 23], including antitumor M1 macrophages and protumor M2 macrophages [16, 24–26]. Large amounts of transmitters, such as M-CSF, IL-6, IL-10, TGF-*β*, and COX-2, induce transformation of TAMs into M2 macrophages that secrete immune inhibitory chemokines with poorer antigen presenting and cytotoxic abilities, leading to tumor growth and metastasis [16, 21, 22, 27–34]. M2 macrophages and TAMs have protumor and immune inhibitory effects, secrete large amounts of IL-10, TGF-*β*, C-C motif chemokine ligand 17 (CCL17), and CCL22, attract noncytotoxic Treg and type II T-helper cells (TH2 cells) to aggregate in tumor tissues, inhibit T-cell differentiation and functions, lower cytotoxic T-cell function, induce T-cell apoptosis, secrete CCL18, and attract naïve T cell [10, 16, 32, 35]. Immune modulatory and antitumor effects of mushroom beta-glucan have been noted by Ikekawa et al. in 1968 in the fruiting body extracts of* Lentinus edodes*,* Coriolus versicolor*,* Ganoderma tsugae*,* Flammulina velutipes,* and* Tricholoma matsutake* which have demonstrated significant antitumor activities towards transplanted tumor cells of sarcoma 180 [36–38]. Celecoxib is a COX-2 inhibitor and inhibits tumor growth by inhibiting synthesis of prostaglandin [39–46]. Nakanishi et al. noted that daily oral administration of celecoxib in tumor-bearing mice (colon cancer) resulted in enhanced secretion of IFN-*γ* by T cells and natural killer cells and altered the immune inhibitory effect in the tumor microenvironment, which in turn induced differentiation of macrophages into M1 macrophages and inhibited tumor growth [47]. We have discussed inflammation materials involved in the carcinogenesis [48]; furthermore in this study, we aim to investigate the mechanism in which mushroom beta-glucan modulates the TAM forward to the M1 macrophages and inhibits M2 macrophages, which in turn adequately inhibits tumor growth and metastasis in this examination.

## 2. Material and Methods

### 2.1. Experimental Protocol

A fixed dose of 1 × 10^6^ cells/50 *μ*L Lewis lung carcinoma cells (LLC1) was administered subcutaneously into the right inner thighs of 5 dependent C57BL/6JNarl mice (National Taiwan University Animal Reproduction and Research Center) for each group, followed by observation of tumor formation at the site of injection after two days. One day after the injection, mice were tube-fed with either twice-distilled water, celecoxib (Pfizer), or mushroom beta-glucan continuously for 12 days.

First, we examine whether the effect of* Antrodia camphorata* beta-glucan modulates the mice physiology response; the experiment group was divided into group 1: PC consisted of normal mice with subcutaneous injection of PBS, followed by oral feed of twice-distilled water; group 2: PB consisted of injection of PBS in normal mice, followed by oral feed of* Antrodia camphorata* beta-glucan. In the following experiment, we researched in the* Antrodia camphorata* beta-glucan involved in modulating the tumor microenvironment using the tumor-bearing mice. The experiment group was divided into group 3: TC consisted of tumor-bearing mice fed with twice-distilled water; group 4: TM consisted of mice fed with celecoxib; group 5: TB consisted of tumor-bearing mice fed with* Antrodia camphorata* beta-glucan; group 6: TX consisted of tumor-bearing mice fed with Ganoderma beta-glucan (Table 2). At day 14, mice were euthanized, and blood samples, tumor tissues, and lungs were collected or harvested. Tumor tissues were weighed, and samples from groups TM, TB, and TX were compared with that of group TC, by calculated tumor inhibitory rate, via ELISA to quantify serum TGF-*β*. RT-qPCR was used to quantify gene expression of IL-12, IFN-*γ*, GM-CSF, M-CSF, IL-6, IL-10, COX-2, and TGF-*β* in lung and tumor tissues. Flow cytometry was used to quantify the percentages of M1 and M2 macrophages in the lungs and tumor tissues.

### 2.2. Mushroom Polysaccharide

Our previous study has examined the safety assessment of mushroom beta-glucan [49]; moreover, in this experiment, mycelium of* Ganoderma lucidum *or* Antrodia camphorata* subcultured and maintained in sterile YM agar (0.02%) was used for the production of MBG. The manufacturing process was initiated by preparing a culture medium containing glucose, lactose, galactose, sucrose, mannose, and yeast extract. Mycelium of* Ganoderma lucidum* or* Antrodia camphorata *was then introduced into the sterile medium and cultured using a shaker incubator at temperatures ranging from 27 to 32°C for 3–5 weeks to achieve a full polymerization of MBG in the culture system. Subsequently, MBG from cultured mycelia was homogenized and disrupted using high speed homogenizer and ultrasonic vibration. The MBG solution was then filtered and concentrated using a ceramic membrane to strip most of the residual small molecules in the solution. The concentrated MBG was dried by lyophilization and then grinded into the powdered form. The sample was demonstrated to contain approximately 95% carbohydrate, 1% fat, 1% protein, 2% of ash, and 0.8% of water. Using Megazyme (Ireland) mushroom and yeast Beta-Glucan Kit, the crude extract was demonstrated to contain approximately 60–65% of MBG (MBG). The molecular weight of MBG was analyzed by high pressure liquid chromatographic (HPLC) using Shodex sugar KS series containing KS-G, KS-804, and KS-805 columns and detected using RI 2000 detector. Molecular weight was determined by referring to the standard cure using standard molecules including STDP-800 (molecular weight M.W. 8 × 10^5^), STDP-400 (M.W. 4 × 10^5^), STDP-200 (M.W. 2 × 10^5^), STDP-100 (M.W. 1 × 10^5^), and STDP-20 (M.W. 2 × 10^4^). MBG was also processed for analysis of its glycosyl-linkage. The sample was premethylated, depolymerized, reduced, and acetylated. The resultant partially methylated alditol acetates (PMAAs) were then analyzed by gas chromatography-mass spectrometry (GC-MS) according to the procedures described by York et al. and Ciucanu and Kerek [50, 51].

Result from the HPLC analysis showed that MBG powder contained high molecular weight particles that ranged from 9.6 to 298 kDa. The result of GC-MS analysis showed that MBG powder contained 2-; 4-; and 6-; linked galactopyranosyl residues and 3-; 4-; 3,4-; 2,4-; 4,6-; and 3,4,6-linked glucopyranosyl residues.

### 2.3. Tumor-Bearing Mice

Lewis lung carcinoma cell (LLC, purchased from BCRC, Taiwan, BCRC #60050) was placed in 15 mL centrifuge tube with the cultured medium (DMEM, Sigma #D5648) and centrifuged at 200 ×g for five minutes. After removal of supernatant, cells were washed with PBS to further remove allergens in cell medium. Cells were then suspended in PBS culture medium at a concentration of 1 × 10^6^ cells/50 *μ*L and injected into the right inner thighs of the mice. Mice were treated, handled, and cared for following the NIH Guide, The Care and Use of Laboratory Animals. Tumor-bearing mice were euthanized prior to occurrence of cancer-associated symptoms that would limit the animal's mobility or normal daily function. After the animals were euthanized, blood samples, tumor tissues, and lungs were collected or harvested.

### 2.4. Serum Concentration of TGF-*β*

Mouse TGF-*β*Platinum ELISA Kit (eBioscience) was used to quantify serum TGF-*β*. Experimental protocol published by Mouse TGF-*β*Platinum ELISA Kit was followed, and samples were further analyzed with BioTek. Enzyme immunoassay analyzer (ELISA) was set at 450 nm.

### 2.5. Real-Time Polymerase Chain Reaction

RT-qPCR was used to analyze gene expressions of IL-12, IFN-*γ*, GM-CSF, M-CSF, IL-6, IL-10, COX-2, and TGF-*β* in the lungs, using *β*-actin as the reference gene. Experiments were conducted following the protocol published by SYBR Green Supermix Kits (Bio-Rad), using Bio-Rad CFX384 touch RT-PCR for analysis (Table 1).

### 2.6. Proportions of M1 and M2 Macrophages in the Lung and Tumor Cells

#### 2.6.1. Centrifuge

Harvested lung and tumor tissues were cut into small pieces and placed in the culture medium and incubated with 5 mL PBS (containing 0.1 mg/mL collagenase (SIGMA)) at 37°C for 30 minutes, followed by passing of the ground tissues through steel mesh with 70 *μ*m. The resulting cell suspension fluid was added into 5 mL Histopaque 1080 (SIGNMA) centrifuge tube and centrifuged for 30 minutes at 4°C, 400 ×g. After removing the supernatant and several rounds of washings with PBS to clear the Histopaque, FACS buffer was added to the cell suspension fluid to obtain final cell counts. Cell suspension fluid concentration was readjusted to 1 × 10^7^ cells/mL and maintained at 4°C for the experiments.

#### 2.6.2. Surface Marker Staining

100 *μ*L of suspension fluid was added to 0.25 *μ*g APC anti-mouse F4/80 antibody (BioLegend), 1.0 *μ*g PE anti-mouse CD86 antibody (BioLegend), and 0.125 *μ*g FITC anti-mouse CD206 antibody (BioLegend), respectively. Homogenized solutions were placed in the dark at 4°C for 30 minutes, followed by addition of 200 *μ*L FACS buffer, and centrifuged at 4°C, 300 ×g for 5 minutes. After removal of supernatant, the cellular solutions were washed with FACS buffer, followed by addition of 1 mL FACS buffer to resuspend cellular samples. Finally, cellular aggregates were broken up and analyzed with BD FACSCanto II, APC anti-mouse F4/80 antibody-specific M1 macrophage (F4/80^+^), APC anti-mouse F4/80 antibody, and FITC anti-mouse CD206 antibody-specific M2 macrophage (F4/80^+^, CD206^+^). FlowJo software was used to analyze the percentage of macrophages in the lung tissues and the proportions of M1 and M2 macrophages in the lung and tumor tissue samples.

Percentage of macrophages: macrophages (F4/80^+^)/cells.

Percentage of M1 macrophages: M1 (F4/80^+^, CD86^+^)/macrophages (F4/80^+^).

Percentage of M2 macrophages: M2 (F4/80^+^, CD206^+^)/macrophages (F4/80^+^).

### 2.7. Statistical Analysis

Analysis was conducted using SPSS 17.0 software. One-way analysis of variance, one-way ANOVA, and Scheffe's method were conducted, with significance set at *P* < 0.05.

## 3. Results

### 3.1. Physiologic Effects of* Antrodia camphorata* Beta-Glucan on Normal Mice

#### 3.1.1. Effect of* Antrodia camphorata* Beta-Glucan on Serum TGF-*β* Quantity in Normal Mice

**Figure 1:**
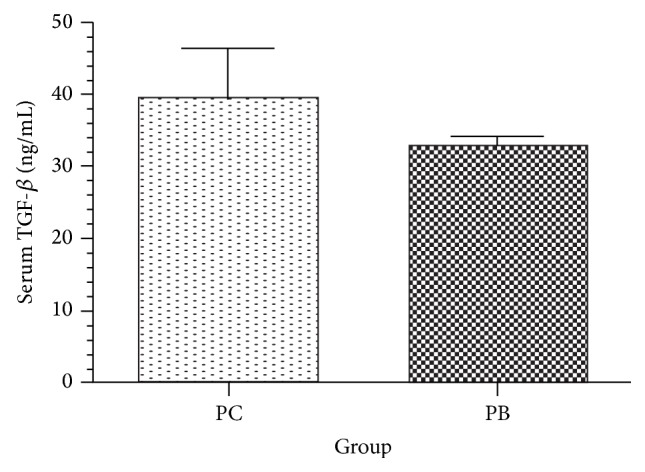
Effect of* Antrodia camphorata* beta-glucan on the amount of serum TGF-*β*. Normal mice were fed with either twice-distilled water or* Antrodia camphorata* beta-glucan daily and euthanized after 12 days. Blood samples were then collected (*n* = 5). ^*^Group PC (control): fed with twice-distilled water; group PB (experiment): fed with* Antrodia camphorata* beta-glucan.

Serum TGF-*β* quantity in PC group (control) was at 39.59 ± 5.645 ng/mL and compared to group PB (experiment) at 32.8 ± 1.879 ng/mL. There is no significant difference between the two groups (*P* > 0.05). Daily oral intake of* Antrodia camphorata* beta-glucan does not alter serum TGF-*β* in normal mice (Figure 1).

#### 3.1.2. Effect of* Antrodia camphorata* Beta-Glucan on Quantities of Lung Cytokines and Gene Expression of Growth Factors in Normal Mice

**Figure 2:**
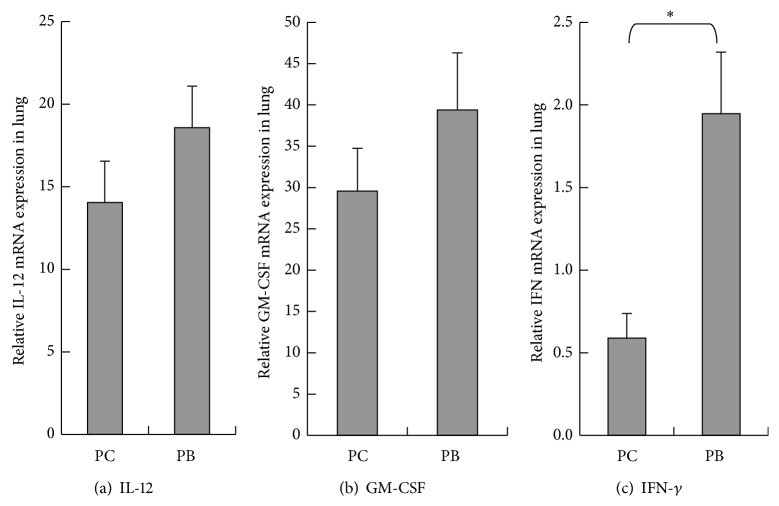
Effect of* Antrodia camphorata* beta-glucan on the amount of gene expressions of IL-12, GM-CSF, and IFN-*γ* in the lungs. Normal mice were fed with either twice-distilled water or* Antrodia camphorata* beta-glucan daily and euthanized after 12 days. Lungs were then harvested (*n* = 5). RT-qPCR was used to analyze gene expressions of IL-12, GM-CSF, and IFN-*γ* in the lungs. *β*-actin was used as the reference gene. (a) Amount of IL-12 gene expression. (b) Amount of GM-CSF gene expression. (c) Amount of IFN-*γ* gene expression. ^*∗*^Group PC: fed with twice-distilled water; group PB: fed with* Antrodia camphorata* beta-glucan.

**Figure 3:**
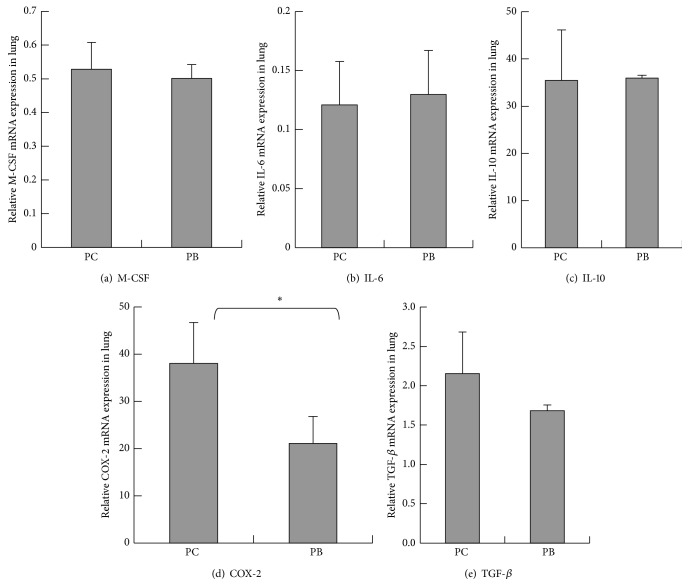
Effect of* Antrodia camphorata* beta-glucan on the amount of gene expressions of M-CSF, IL-6, IL-10, COX-2, and TGF-*β* in the lungs. Normal mice were fed with distilled water,* Antrodia camphorata* beta-glucan, or Ganoderma beta-glucan daily and euthanized after 12 days. Lungs were then harvested (*n* = 5). RT-qPCR was used to quantify the gene expressions of M-CSF, IL-6, IL-10, COX-2, and TGF-*β* in the lungs. *β*-actin was used as the reference gene. (a) Amount of M-CSF gene expression. (b) Amount of IL-6 gene expression. (c) Amount of IL-10 gene expression. (d) Amount of COX-2 gene expression. (e) Amount of TGF-*β* gene expression. ^*∗*^Group PC: fed with twice-distilled water; group PB: fed with* Antrodia camphorata* beta-glucan.

**Figure 4:**
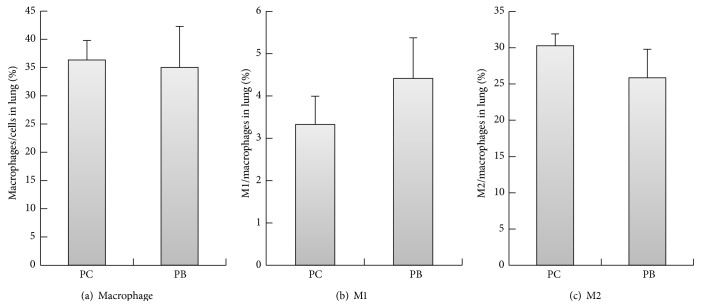
Effect of* Antrodia camphorata* beta-glucan on the percentages of macrophages and proportions of M1 and M2 macrophages. Normal mice were fed with distilled water or* Antrodia camphorata* beta-glucan daily and euthanized after 12 days. Lungs were then harvested (*n* = 5). Flow cytometer was used to analyze the percentages of M1 and M2 macrophages in the lungs. (a) Percentages of macrophages. (b) Percentages of M1 macrophages. (c) Percentages of M2 macrophages. ^*^Group PC: fed with twice-distilled water; group PB: fed with* Antrodia camphorata* beta-glucan.

As results shown in Figure 2(a), the amounts of IL-12 gene expression in the lungs of normal mice are as follows: group PB is at 0.0019 ± 0.00025, and group PC is lower at 0.0014 ± 0.00025. There is no statistical significant difference between groups PC and PB (*P* > 0.05). As results shown in Figure 2(b), the amounts of GM-CSF gene expression in the lungs of normal mice are as follows: group PB is at 39.3786 ± 6.90311,, and group PC is at 29.5757 ± 5.17426. There is no statistical significant difference between groups PC and PB (*P* > 0.05). As results shown in Figure 2(c), the amounts of IFN-*γ* gene expression in the lungs of normal mice are as follows: group PB is at 1.9469 ± 0.37199, and group PC is at 0.5895 ± 0.14802. The quantity of IFN-*γ* gene expression of group PB is statistically significantly higher than that of group PC (*P* < 0.05). As results shown in Figure 3(a), the amounts of M-CSF gene expression in the lungs of normal mice are as follows: group PC is higher at 0.5285 ± 0.07916, and group PB is lower at 0.5012 ± 0.04078. There is no statistical significant difference between groups PC and PB. As results shown in Figure 3(b), the amounts of IL-6 gene expression in the lungs of normal mice are as follows: group PB is higher at 0.1297 ± 0.03755, and group PC is lower at 0.1208 ± 0.03685. There is no statistical significant difference between groups PC and PB. As results shown in Figure 3(c), the amounts of IL-10 gene expression in the lungs of normal mice are as follows: group PB is higher at 0.0036 ± 0.00005, and group PC is lower at 0.0035 ± 0.00107. There is no statistical significant difference between groups PC and PB. As results shown in Figure 3(d), the amounts of COX-2 gene expression in the lungs of normal mice are as follows: group PC is higher at 0.0038 ± 0.00087, and group PB is lower at 0.0021 ± 0.00057. The quantity of COX-2 gene expression of group PB is statistically significantly lower than PC group (*P* < 0.05). As results shown in Figure 3(e), the amounts of TGF-*β* gene expression in the lungs of normal mice are as follows: group PC is higher at 2.1539 ± 0.5294, and group PB is lower at 1.6817 ± 0.07353. There is no statistical significant difference between groups PC and PB. Results shown in Figure 4(a) demonstrated higher group PC value at 36.32 ± 3.458% and lower group PB value at 35 ± 7.291%. There is no statistical significant difference between groups PC and PB in the lung M1 macrophage percentages. As results shown in Figure 4(b), percentages of M1 macrophages in the lung tissues of normal mice are higher in PB group at 4.41 ± 0.956% and lower in group PC at 3.33 ± 0.668%. There is no statistical significant difference between groups PC and PB. As results shown in Figure 4(c) for the percentages of M2 macrophage in the lung tissues, group PC is higher at 30.28 ± 1.612%, and group PB is lower at 25.86 ± 3.95%. There is no statistical significant difference between groups PC and PB.

### 3.2. Effect of Mushroom Beta-Glucan on Tumor-Bearing Mice

#### 3.2.1. Effect of Mushroom Beta-Glucan on Serum TGF-*β* Quantity in Tumor-Bearing Mice

**Figure 5:**
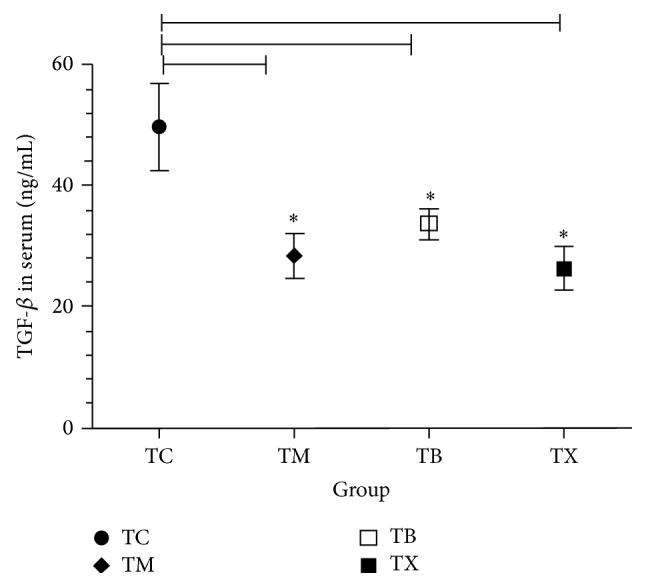
Effect of* Antrodia camphorata* beta-glucan on the amount of serum TGF-*β* in tumor-bearing mice. Tumor-bearing mice were fed with either twice-distilled water, celecoxib,* Antrodia camphorata* beta-glucan, or Ganoderma beta-glucan daily and euthanized after 12 days. Blood samples were then collected (*n* = 5). TGF-*β* Platinum ELISA Kit was used to quantify amount of serum TGF-*β*. ^*∗*^Group TC: fed with twice-distilled water; group TM: fed with celecoxib; group TB: fed with* Antrodia camphorata* beta-glucan; group TX: fed with Ganoderma beta-glucan.

As results shown in Figure 5, amount of serum TGF-*β* in tumor-bearing mice is shown to be higher in group TC at 49.8 ± 12.454 ng/mL, followed by group TB at 33.64 ± 4.045 ng/mL and group TM at 28.45 ± 6.274 ng/mL, and lowest in group TX at 26.33 ± 5.901 ng/mL. Groups TM, TB, and TX show significantly lower serum TGF-*β* amount than group TC (*P* < 0.05). However, there are no statistically significant differences between groups TB, TX, and TM. Results show that daily oral intake of celecoxib or* Antrodia camphorata* beta-glucan in tumor-bearing mice lowers the amount of serum TGF-*β* in tumor-bearing mice.

#### 3.2.2. Effect of Mushroom Beta-Glucan on Quantities of Lung Cytokines and Gene Expression of Growth Factors in Tumor-Bearing Mice

**Figure 6:**
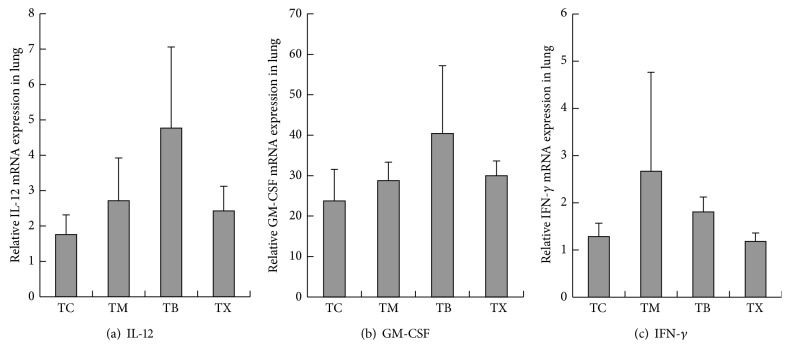
Effect of* Antrodia camphorata* beta-glucan on the gene expressions of IL-12, GM-CSF, and IFN-*γ* in the lungs of tumor-bearing mice. Tumor-bearing mice were fed with either twice-distilled water, celecoxib,* Antrodia camphorata* beta-glucan, or Ganoderma beta-glucan daily and euthanized after 12 days. Lungs were then harvested (*n* = 5). RT-qPCR was used to quantify the amount of gene expressions of IL-12, GM-CSF, and IFN-*γ*. ^*∗*^Group TC: fed with twice-distilled water; group TM: fed with celecoxib; group TB: fed with* Antrodia camphorata* beta-glucan; group TX: fed with Ganoderma beta-glucan.

**Figure 7:**
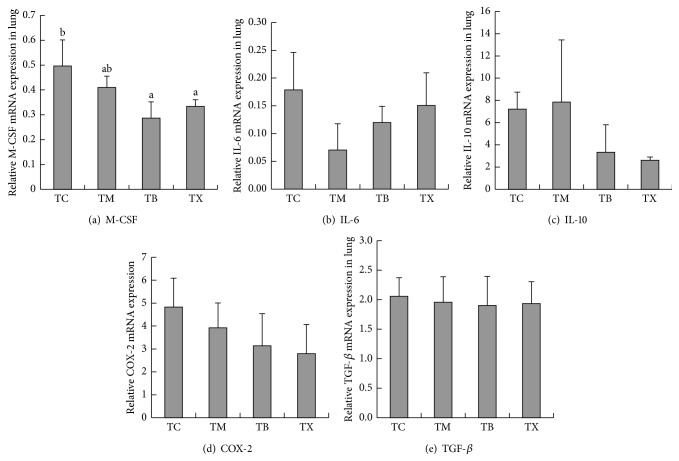
Effect of Ganoderma beta-glucan on the gene expressions of M-CSF, IL-6, IL-10, COX-2, and TGF-*β* in the lungs of tumor-bearing mice. Tumor-bearing mice were fed with either twice-distilled water, celecoxib,* Antrodia camphorata* beta-glucan, or Ganoderma beta-glucan daily and euthanized after 12 days. Lungs were then harvested (*n* = 5). RT-qPCR was used to quantify the amount of gene expressions of M-CSF, IL-6, IL-10, COX-2, and TGF-*β* in the lungs. *β*-actin was used as the reference gene. (a) Amount of M-CSF gene expression. (b) Amount of IL-6 gene expression. (c) Amount of IL-10 gene expression. (d) Amount of COX-2 gene expression. (e) Amount of TGF-*β* gene expression. ^*∗*^Group TC: fed with twice-distilled water; TM: fed with celecoxib; group TB: fed with* Antrodia camphorata* beta-glucan; group TX: fed with Ganoderma beta-glucan.

As results shown in Figure 6(a), the amounts of IL-12 gene expression in the lungs of tumor-bearing mice are the highest in group TB at 0.0048 ± 0.0023, followed by group TM at 0.0027 ± 0.00121 and group GX at 0.0024 ± 0.0007, and the lowest in group TC at 0.0018 ± 0.00056. There are no statistically significant differences between groups TM, TB, and TX. As results shown in Figure 6(b), the amounts of GM-CSF gene expression in the lungs of tumor-bearing mice are shown to be the highest in group TB at 40.4202 ± 16.8, followed by group TX at 29.9667 ± 3.67509 and group TM at 28.7425 ± 4.58706, and the lowest in group TC at 23.7612 ± 7.77548. There are no statistically significant differences between groups TM, TB, TX, and TC. As results shown in Figure 6(c), the amounts of IFN-*γ* gene expression in the lungs of tumor-bearing mice are the highest in group TM at 2.6666 ± 2.10062, followed by group TB at 1.8043 ± 0.31818 and group TC at 1.2801 ± 0.28564, and the lowest in group TX at 1.1816 ± 0.17898. There are no statistically significant differences between groups TM, TB, TX, and TC. As results shown in Figure 7(a), the amounts of M-CSF gene expression in the lungs of tumor-bearing mice are the highest in group TC at 0.4965 ± 0.1044, followed by group TM at 0.4098 ± 0.0458 and group TX at 0.3341 ± 0.02674, and the lowest in group TB at 0.2865 ± 0.06564. The amounts of M-CSF gene expression in the lungs of tumor-bearing mice are statistically significantly lower in groups TB and TX when compared to group TM but not statistically significantly different when compared to groups TC and TM. As results shown in Figure 7(b), the amounts of IL-6 gene expression in the lungs of tumor-bearing mice are the highest in group TC at 0.1788 ± 0.06732, followed by group TX at 0.1505 ± 0.05891 and group TB at 0.1199 ± 0.02936, and the lowest in group TM at 0.0705 ± 0.04733. There are no statistically significant differences between groups TM, TB, TX, and TC. As result shown in Figure 7(c), the amounts of IL-10 gene expression in the lungs of tumor-bearing mice are the highest in group TM at 0.0079 ± 0.00559, followed by group TC at 0.0072 ± 0.00153 and group TB at 0.0033 ± 0.00246, and the lowest in group TX at 0.0026 ± 0.0003. As results shown in Figure 7(d), the amounts of COX-2 gene expression in the lungs of tumor-bearing mice are the highest in group TC at 0.0048 ± 0.00127, followed by group TM at 0.0039 ± 0.00108 and group TB at 0.0031 ± 0.00141, and the lowest in group TX at 0.0028 ± 0.00128. There are no statistically significant differences between groups TM, TB, TX, and TC. As results shown in Figure 7(e), the amounts of TGF-*β* gene expression in the lungs of tumor-bearing mice are the highest in group TC at 2.058 ± 0.31498, followed by group TM at 1.9589 ± 0.43123 and group TX at 1.9361 ± 0.36897, and the lowest in group TB at 1.9032 ± 0.49164. There are no statistically significant differences between groups TM, TB, TX, and TC. Composite results show that daily oral intake of mushroom beta-glucan in tumor-bearing mice can lower the amounts of M-CSF gene expression in the lungs but does not affect the amounts of IL-12, GM-CSF, IFN-*γ*, IL-6, IL-10, COX-2, and TGF-*β* in the lungs.

#### 3.2.3. Effect of Mushroom Beta-Glucan on Lung Macrophages and Proportions of M1 and M2 Macrophages in Tumor-Bearing Mice

**Figure 8:**
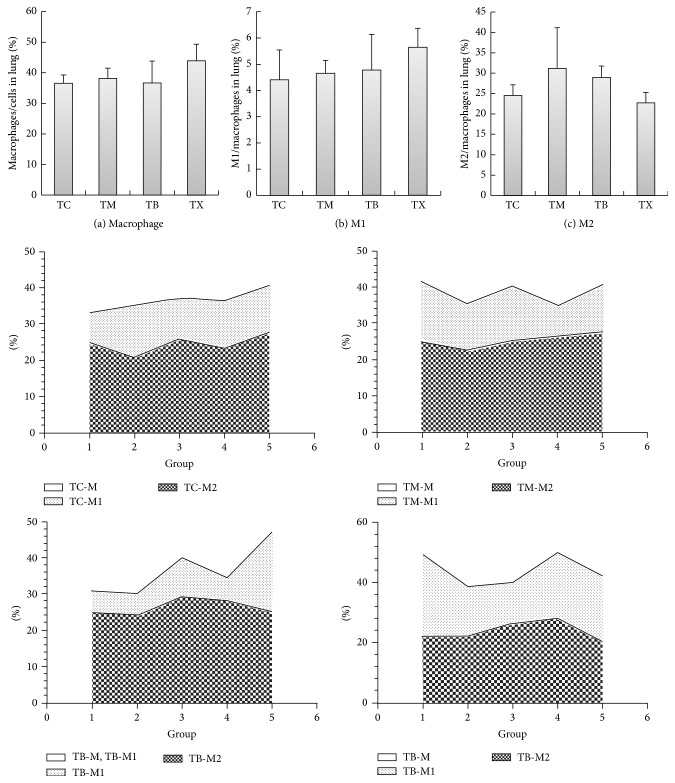
Effect of* Antrodia camphorata* beta-glucan on the percentages of macrophages and proportions of M1 and M2 macrophages in the lungs of tumor-bearing mice. Tumor-bearing mice were fed with either twice-distilled water, celecoxib,* Antrodia camphorata* beta-glucan, or Ganoderma beta-glucan daily and euthanized after 12 days. Lungs were then harvested (*n* = 5). Flow cytometer was used to analyze the percentages of macrophages and proportions of M1 and M2 macrophages in the lungs. (a) Percentages of macrophages. (b) Percentages of M1 macrophages. (c) Percentages of M2 macrophages. ^*^Group TC: fed with twice-distilled water; TM: fed with celecoxib; group TB: fed with* Antrodia camphorata* beta-glucan; group TX: fed with Ganoderma beta-glucan.

As results shown in Figure 8(a), the percentages of macrophages in lung tissues of tumor-bearing mice are the highest in group TX at 43.94 ± 5.396%, followed by group TM at 38.15 ± 3.385% and group TB at 36.66 ± 7.19%, and the lowest in group TC at 36.56 ± 2.753%. There are no statistically significant differences between groups TM, TB, TX, and TC. As results shown in Figure 8(b), the percentages of M1 macrophages in lung tissues of tumor-bearing mice are the highest in group TX at 5.64 ± 0.734%, followed by group TB at 4.77 ± 1.364% and group TM at 4.66 ± 0.493%, and the lowest in group TC at 4.41 ± 1.142%. There are no statistically significant differences between groups TM, TB, TX, and TC. As results shown in Figure 8(c), the percentages of M2 macrophages in lung tissues of tumor-bearing mice are the highest in group TM at 31.17 ± 9.989%, followed by group TB at 28.98 ± 2.766% and group TC at 24.54 ± 2.621%, and the lowest in group TX at 22.73 ± 2.538%. There are no statistically significant differences between groups TM, TB, TX, and TC.

#### 3.2.4. Effect of Mushroom Beta-Glucan on Tumor Tissue Cytokines and Gene Expression of Growth Factors in Tumor-Bearing Mice

**Figure 9:**
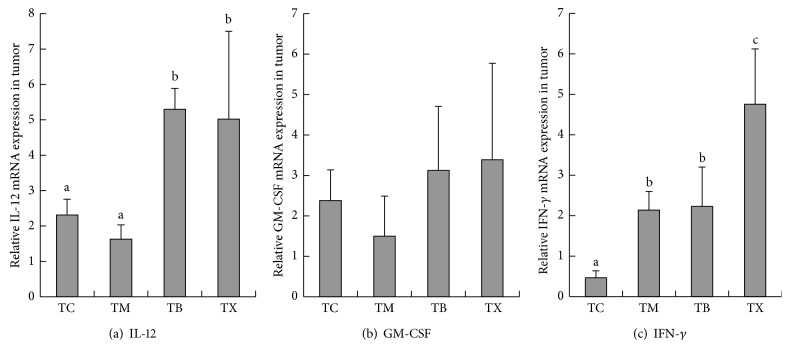
Effect of* Antrodia camphorata* beta-glucan on the amount of gene expressions of IL-12, GM-CSF, and IFN-*γ* in the tumor microenvironment of tumor-bearing mice. Tumor-bearing mice were fed with either twice-distilled water, celecoxib,* Antrodia camphorata* beta-glucan, or Ganoderma beta-glucan daily and euthanized after 12 days. Tumor tissues were then harvested (*n* = 5). RT-qPCR was used to quantify gene expressions of IL-12, GM-CSF, and IFN-*γ* in the tumor microenvironment. *β*-actin was used as the reference gene. ^*∗*^Group TC: fed with twice-distilled water; TM: fed with celecoxib; group TB: fed with* Antrodia camphorata* beta-glucan; group TX: fed with Ganoderma beta-glucan.

**Figure 10:**
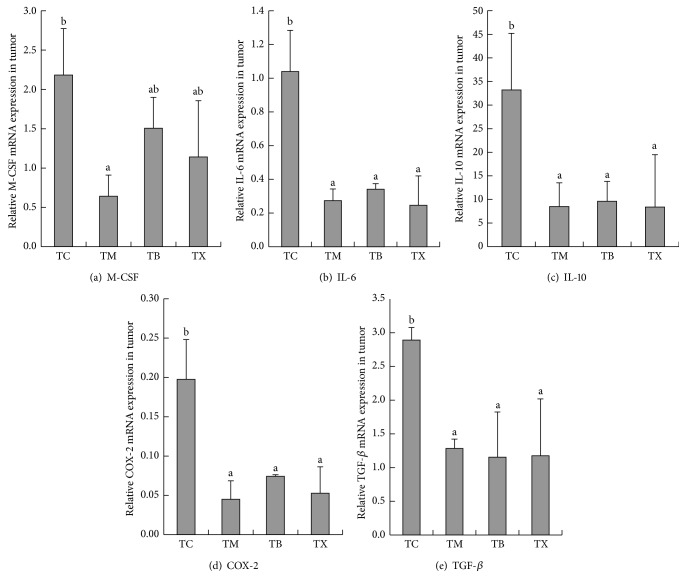
Effect of* Antrodia camphorata* beta-glucan on the amount of gene expressions of M-CSF, IL-6, IL-10, COX-2, and TGF-*β* in the tumor microenvironment of tumor-bearing mice. Tumor-bearing mice were fed with either twice-distilled water, celecoxib,* Antrodia camphorata* beta-glucan, or Ganoderma beta-glucan daily and euthanized after 12 days. Tumor tissues were then harvested (*n* = 5). RT-qPCR was used to quantify gene expressions of M-CSF, IL-6, IL-10, COX-2, and TGF-*β*. *β*-actin was used as the reference gene. (a) Amount of M-CSF gene expression. (b) Amount of IL-6 gene expression. (c) Amount of IL-10 gene expression. (d) Amount of COX-2 gene expression. (e) Amount of TGF-*β* gene expression. ^*∗*^Group TC: fed with twice-distilled water; TM: fed with celecoxib; group TB: fed with* Antrodia camphorata* beta-glucan; group TX: fed with Ganoderma beta-glucan.

As results shown in Figure 9(a), the amounts of IL-12 gene expression in tumor tissues of tumor-bearing mice are the highest in group TX at 0.005 ± 0.0025, followed by group TB at 0.0053 ± 0.00059 and group TC at 0.0023 ± 0.00044, and the lowest in group TM at 0.0016 ± 0.0004. The amounts of IL-12 gene expression in tumor tissues are statistically significantly higher in groups TB and TX when compared to groups TC and TM (*P* < 0.05). However, there is no statistical difference between groups TM and TC. As results shown in Figure 9(b), the amounts of GM-CSF gene expression in tumor tissues of tumor-bearing mice are the highest in group TX at 3.3869 ± 2.38866, followed by group TB at 3.1262 ± 1.58598 and group TC at 2.377 ± 0.76023, and the lowest in group TM at 1.4979 ± 0.99286. There are no statistical differences between groups TM, TB, TX, and TC. As results shown in Figure 9(c), the amounts of IFN-*γ* gene expression in tumor tissues of tumor-bearing mice are the highest in group TX at 4.755 ± 1.37064, followed by group TB at 2.2302 ± 0.97283 and group TM at 2.1372 ± 0.46061, and the lowest in group TC at 0.4663 ± 0.16811. The amounts of IFN-*γ* gene expression in tumor tissues of tumor-bearing mice are statistically significantly higher in groups TM, TB, and TX when compared to group TC (*P* < 0.05), and group TX was statistically significantly higher in group TX than TM (*P* < 0.05). As results shown in Figure 10(a), the amounts of M-CSF gene expression in tumor tissues of tumor-bearing mice are the highest in group TC at 2.1827 ± 0.59147, followed by group TB at 1.5069 ± 0.39195 and group TX at 1.1425 ± 0.71354, and the lowest in group TM at 0.6412 ± 0.2704. The amounts M-CSF gene expression in tumor tissues of tumor-bearing mice are statistically significantly lower in group TM when compared to group TC (*P* < 0.05). However, there are no statistical differences between groups TB, TX, and TC. As results shown in Figure 10(b), the amounts of IL-6 gene expression in tumor tissues of tumor-bearing mice are the highest in group TC at 1.0398 ± 0.24445, followed by group TB at 0.3408 ± 0.03372 and group TM at 0.2742 ± 0.06911, and the lowest in group TX at 0.2463 ± 0.17439. The amounts IL-6 gene expression in tumor tissues of tumor-bearing mice are statistically significantly lower in groups TM, TB, and TX when compared to group TC (*P* < 0.05). However, there are no statistical differences between groups TB, TX, and TM. As results shown in Figure 10(c), the amounts of IL-10 gene expression in tumor tissues of tumor-bearing mice were demonstrated to be the highest in group TC at 0.0332 ± 0.01199, followed by group TB at 0.0096 ± 0.0042 and group TM at 0.0085 ± 0.00505, and lowest in group TX at 0.0084 ± 0.01108. The amounts IL-10 gene expression in tumor tissues of tumor-bearing mice are statistically significantly lower in groups TM, TB, and TX when compared to group TC (*P* < 0.05). However, there are no statistical differences between groups TB, TX, and TM. As results shown in Figure 10(d), the amounts of COX-2 gene expression in tumor tissues of tumor-bearing mice are the highest in group TC at 0.1975 ± 0.05064, followed by group TB at 0.0742 ± 0.00202 and group TX at 0.0527 ± 0.03357, and the lowest in group TM at 0.045 ± 0.02347. The amounts COX-2 gene expression in tumor tissues of tumor-bearing mice are statistically significantly lower in groups TM, TB, and TX when compared to group TC (*P* < 0.05). However, there are no statistical differences between groups TB, TX, and TM. As results shown in Figure 10(e), the amounts of TGF-*β* gene expression in tumor tissues of tumor-bearing mice are the highest in group TC at 2.8931 ± 0.18312, followed by group TM at 1.2829 ± 0.13795 and group TX at 1.1744 ± 0.8451, and the lowest in group TB at 1.1555 ± 0.66985. The amounts of TGF-*β* gene expression in tumor tissues of tumor-bearing mice are statistically significantly lower in groups TM, TB, and TX when compared to group TC (*P* < 0.05). However, there are no statistical differences between groups TB, TX, and TM. Composite results show that daily oral intake of celecoxib in tumor-bearing mice can lower the amounts of M-CSF, IL-6, IL-10, COX-2, and TGF-*β* gene expression in tumors of tumor-bearing mice. Daily oral intake of mushroom beta-glucan in tumor-bearing mice increases the amounts of IL-12 and IFN-*γ* gene expression and lowers the gene expression of IL-6, IL-10, COX-2, and TGF-*β* in tumor tissues of tumor-bearing mice.

#### 3.2.5. Effect of Mushroom Beta-Glucan on TAMs and Proportions of M1 and M2 Macrophages in Tumor Tissue in Tumor-Bearing Mice

**Figure 11:**
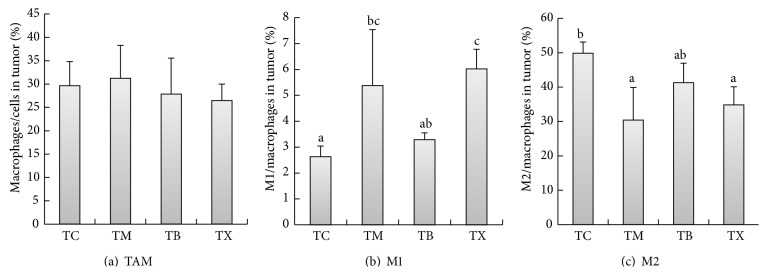
Effect of* Antrodia camphorata* beta-glucan on the percentages of macrophages and proportions of M1 and M2 macrophages in the tumor microenvironment of tumor-bearing mice. Tumor-bearing mice were fed with distilled water, celecoxib,* Antrodia camphorata* beta-glucan, or Ganoderma beta-glucan daily and euthanized after 12 days. Tumor tissue samples were then harvested (*n* = 5). Flow cytometer was used to analyze percentages of macrophages and proportions of M1 and M2 macrophages in the tumor microenvironment. (a) Percentages of macrophages in the tumor. (b) Percentages of M1 macrophages in the tumor. (c) Percentages of M2 macrophages in the tumor. ^*∗*^Group TC: fed with twice-distilled water; group TM: fed with celecoxib; group TB: fed with* Antrodia camphorata* beta-glucan; group TX: fed with Ganoderma beta-glucan.

As results shown in Figure 11(a), the percentages of macrophages in tumors of tumor-bearing mice are the highest in group TM at 31.23 ± 7.056%, followed by group TC at 29.64 ± 5.186% and group TB at 27.84 ± 7.739%, and the lowest in group TX at 26.46 ± 3.546%. There are no statistical differences between groups TM, TB, TX, and TC. As results shown in Figure 11(b), the percentages of M1 macrophages in tumors of tumor-bearing mice are the highest in group TX at 6.02 ± 0.759%, followed by group TM at 5.38 ± 2.168% and group TB at 3.29 ± 0.262%, and the lowest in group TC at 2.63 ± 0.412%. The percentages of M1 macrophages in tumors of tumor-bearing mice are statistically significantly higher in groups TM and TX when compared to group TC (*P* < 0.05). However, there is no statistical difference between groups TB and TC. As results shown in Figure 11(c), the percentages of M2 macrophages in tumors of tumor-bearing mice are the highest in group TC at 49.85 ± 3.297%, followed by group TB at 41.27 ± 5.689% and group TX at 34.83 ± 5.254%, and the lowest in group TM at 30.4 ± 9.496%. The percentages of M2 macrophages in tumors of tumor-bearing mice are statistically significantly lower in groups TM and TX when compared to group TC (*P* < 0.05). However, there is no statistical difference between groups TB and TC. Composite results show that daily oral intake of celecoxib or mushroom beta-glucan in tumor-bearing mice can increase the percentage of M1 macrophages and lower the percentage of M2 macrophages in tumor-bearing mice.

## 4. Discussions

Nakanishi et al. found that celecoxib can alter the immune inhibitory effects of the tumor microenvironment by promoting transformation of TAMs into M1 macrophages, leading to inhibited tumor growth [47]. In our study, control group consisted of mice fed with celecoxib. After subcutaneous injection of LLC1 tumor cells and tumor development at the injection site, tumor-bearing mice were tube-fed with distilled water (group TC), celecoxib (group TM),* Antrodia camphorata*-derived beta-glucan (group TB), or* Ganoderma lucidum*-derived beta-glucan (group TX), respectively, for 12 consecutive days, and tumor sizes were recorded. Our study found that oral intake of celecoxib slowed tumor growth by 48.15%. In 1968, Ikekawa et al. found that the fruiting body extracts from* Lentinus edodes*,* Trametes versicolor*,* Ganoderma tsugae*,* Flammulina velutipes,* and* Tricholoma matsutake* demonstrated significant antitumor activities towards transplanted tumor cells of sarcoma 180 [36, 37, 52, 53].

In previous studies,* Antrodia camphorata*-derived beta-glucan has demonstrated inhibitory effects on tumor growth for sarcoma 37, sarcoma 180, Ehrlich ascites sarcoma, Yoshida sarcoma, and LLC1 transplanted tumor growth [54]. Daily intake of* Antrodia camphorata*-derived beta-glucan for 18 consecutive days has been demonstrated to slow tumor growth and reduce the rate of metastasis [55]. Cytotoxic T-cells activity and tumor occurrence rate were observed. Results showed that daily oral intake of* Grifola frondosa*-derived beta glucan or Lentinan can enhance cytotoxic T-cells activity and decrease tumor occurrence rate [56]. Additionally, they found that the addition of conditioned medium with tumor cells into the progenitor cells of dendritic cells can further inhibit maturation of dendritic cells and lower the antigen presenting capability of the dendritic cells [57]. Tumor cells were found to secrete M-CSF, inhibiting dendritic and T-cell differentiation and antitumor abilities [1, 57–59]. Our studies found that daily oral intake of mushroom beta-glucan from* Antrodia camphorata *or* Ganoderma lucidum* in tumor-bearing mice can reduce the amount of M-CSF gene expression in the lungs and that daily oral intake of celecoxib in tumor-bearing mice can reduce the amount of M-CSF gene expression in the tumor tissues.

The presented research has indicated that anticancer drugs are generally plagued by toxic manifestations at doses necessary for control of various forms of cancer; in order to alternate the side effect of the anticancer drug, some antioxidants and immunomodulators such as tuftsin [60, 61], picroliv [62], and medical mushroom [63, 64] have also been applied to impart significant antitumor activity presumably by nonspecific activation of the host immune system [65]. In this presented research, we exactly to examine the polysaccharide from medical mushroom to apply in the cancer therapy, and the data shown that the observation of the inflammation and toxicity response not to be significantly presented while feeding with the polysaccharide in the normal (control) group, but the function of anticancer by immunomodulation was to be observed in the tumor-bearing mice. We preliminarily conclude that daily oral intake of mushroom beta-glucan from* Antrodia camphorata *and* Ganoderma lucidum* in tumor-bearing mice can reduce the amount of M-CSF gene expression in the lungs. Daily oral intake of mushroom beta-glucan from* Antrodia camphorata* and* Ganoderma lucidum* in tumor-bearing mice can reduce the amount of M-CSF gene expression and in turn enhance differentiation of dendritic cells and their antigen presenting ability. Daily oral intake of celecoxib in tumor-bearing mice can lower the amount of gene expression of M-CSF by the tumor tissues, enhance differentiation of dendritic cell and T cells, and in turn reduce the immune-inhibitory effect of the tumor environment and inhibit the immune inhibitory effect of the tumor environment, further inhibiting tumor growth. In the tumor environment, the amounts of M1 and M2 macrophages are not equal [66]. It is currently known that tumor environment contains large amount of transmitters such as M-CSF, IL-6, IL-10, TGF-*β*, and COX-2 that induces tumor megakaryocytes to differentiate into M2 macrophages, which, in addition to having poorer antigen-presenting and cytotoxic abilities, also secretes factors that inhibit immune cells, resulting in enhanced immune inhibitory effect of the tumor environment [16, 21, 22, 27–34]. M2 macrophage in the tumor-bearing mice enhances tumor growth and immune inhibitory effects. They also secrete cytokines, such as IL-10 and TGF-*β*, in high quantities, that attract noncytotoxic Treg-cells and type 2 helper T cells to congregate in tumor tissues, which in turn inhibit the differentiation and normal functions of T cells, including their cytotoxic ability, and further lead to T-cells apoptosis [16, 32, 35, 67, 68].

Our study found that daily oral intake of celecoxib or mushroom beta-glucan from* Antrodia camphorata* can decrease the gene expression of IL-6, IL-10, COX-2, and TGF-*β* and further decreases the proportion of M2 macrophages in tumor-bearing mice. Based on these results, oral intake of celecoxib or mushroom beta-glucan from* Antrodia camphorata* can decrease the gene expression of IL-6, IL-10, COX-2, and TGF-*β* and further decreases the proportion of M2 macrophages in tumor-bearing mice, as well as decreasing the secretion of cytokines, such as IL-10 and TGF-*β* that decreases the immune inhibitory effect in the tumor environment.

## 5. Conclusion

Oral intake of mushroom beta-glucan in tumor-bearing mice demonstrated an increase in the gene expression of IL-12 and IFN-*γ* in tumor tissues and a decrease in serum TGF-*β* concentration and gene expressions of IL-6, IL-10, COX-2, and TGF-*β* in the tumor microenvironment. Our study found that mushroom beta-glucan can reduce the immune inhibitory effects of the tumor microenvironment in the host. Alteration of the tumor microenvironment promotes transformation of TAMs into M1 macrophages and reduces the transformation of TAMs into M2 macrophages.

## Supplementary Material

Supplement Material 1: The observation of the tumor size after oral the mushroom beta glucan compared to the control (fed with twice-distilled water).
Supplement Material 2: Using the HPLC to analyze the *Genoderma lucidum* polysaccharides.
Supplement Material 3: Using the HPLC to analyze the *Ganoderma lucidum* polysaccharides.
Supplement Material 4: The Flow cytometry analyze the percentage of the M1 and M2 ration in each group.

## Figures and Tables

**Table 1:** Primer sequence.

Target Gene	GenBank #	Forward sequence (5′ to 3′)	Reverse sequence (5′ to 3′)
*β*-actin	BC138614.1	AATCGTGCGTGACATCAA	AGAAGGAAGGCTGGAAAA
IFN-*γ*	BC119063.1	TCTGAGACAATGAACGCTAC	TTCCACATCTATGCCACT
GM-CSF	BC116880.1	GAAGATATTCGAGCAGGGTC	GAAATCCGCATAGGTGGT
IL-12	M86671.1	TGAAAGGCTGGGTATCGG	GCTGGAACTCCCTCTGTA
COX-2	BC052900.1	ATGACTGCCCAACTCCCA	AACCCAGGTCCTCGCTTA
IL-6	BC138766.1	TGCCTTCTTGGGACTGAT	TTGCCATTGCACAACTCTTT
M-CSF	M21149.1	TTCTACAAGTGGAAGTGGAGG	AGAGGGACATTGACAAACG
IL-10	BC137844.1	TTTCAAACAAAGGACCAG	GGATCATTTCCGATAAGG
TGF-*β*	M13177.1	GGCGGTGCTCGCTTTGTA	TTTCTCATAGATGGCGTTGTT

**Table 2:** Figure out the parameter detected in the presented study.

	Normal mice		Tumor-bearing mice			
	PC (distilled water)	PB (fed with Ganoderma beta-glucan)	TC (fed with distilled water)	TM (fed with celecoxib)	TB (fed with Ganoderma beta-glucan)	TX (fed with Ganoderma beta-glucan)
TGF-*β* (serum)	—	—	—	↓	↓	↓

IL-12 (lung)	—	—	—	—	—	—

IL-12 (tumor)	*✗*	*✗*	—	—	↑	↑

GM-CSF (lung)	—	—	—	—	—	—

GM-CSF (tumor)	*✗*	*✗*	—	—	—	—

IFN-*γ* (lung)	—	↑	—	—	—	—

IFN-*γ* (tumor)	*✗*	*✗*	—	↑	↑	↑

IL-10 (lung)	—	—	—	—	—	—

IL-10 (tumor)	*✗*	*✗*	—	↓	↓	↓

M-CSF (lung)	—	—	—	—	↓	↓

M-CSF (tumor)	*✗*	*✗*	—	↓	—	—

TGF-*β* (lung)	—	—	—	—	—	—

TGF-*β* (tumor)	*✗*	*✗*	—	↓	↓	↓

IL-6 (lung)	—	—	—	—	—	—

IL-6 (tumor)	*✗*	*✗*	—	↓	↓	↓

COX-2 (lung)	—	↓	—	—	—	—

COX-2 (tumor)	*✗*	*✗*	—	↓	↓	↓
