# Supplementary material for: Mushroom β-Glucan May Immunomodulate the Tumor-Associated Macrophages in the Lewis Lung Carcinoma
Source: Biomed Res Int. 2015 Jun 17;2015:604385. doi: 10.1155/2015/604385 (PMC4488085; doi:10.1155/2015/604385)
Supplement: Supplementary file 1 — Supplement Material 1: The observation of the tumor size after oral the mushroom beta glucan compared to the control (fed with twice-distilled water). Supplement Material 2: Using the HPLC to analyze the Genoderma lucidum polysaccharides. Supplement Material 3: Using the HPLC to analyze the Ganoderma lucidum polysaccharides. Supplement Material 4: The Flow cytometry analyze the percentage of the M1 and M2 ration in each group. [file 604385.f1.pdf]

Supplement Data

Legends

Supplement Data 1. The observation of the tumor size after oral the mushroom beta glucan compared to the control (fed with twice-distilled water). *Group TC: fed with twice-distilled water; TM: fed with celecoxib; group TB: fed with *Antrodia camphorata* beta-glucan; group TX: fed with Ganoderma beta-glucan.

Supplement Data 2. Using the HPLC to analyze the *Genoderma lucidum* polysaccharides

Supplement Data 3. Using the HPLC to analyze the *Ganoderma lucidum* polysaccharides

Supplement Data 4. The Flow cytometry analyze the percentage of the M1 and M2 ration in each group. *Group PC: fed with twice-distilled water; group PB: fed with *Antrodia camphorata* beta-glucan (Without Tumor Bearing). Group TC: fed with twice-distilled water; group TM: fed with celecoxib; group TB: fed with *Antrodia camphorata* beta-glucan; group TX: fed with Ganoderma beta-glucan. (Tumor Bearing)

Supplement Data 1. The observation of the tumor

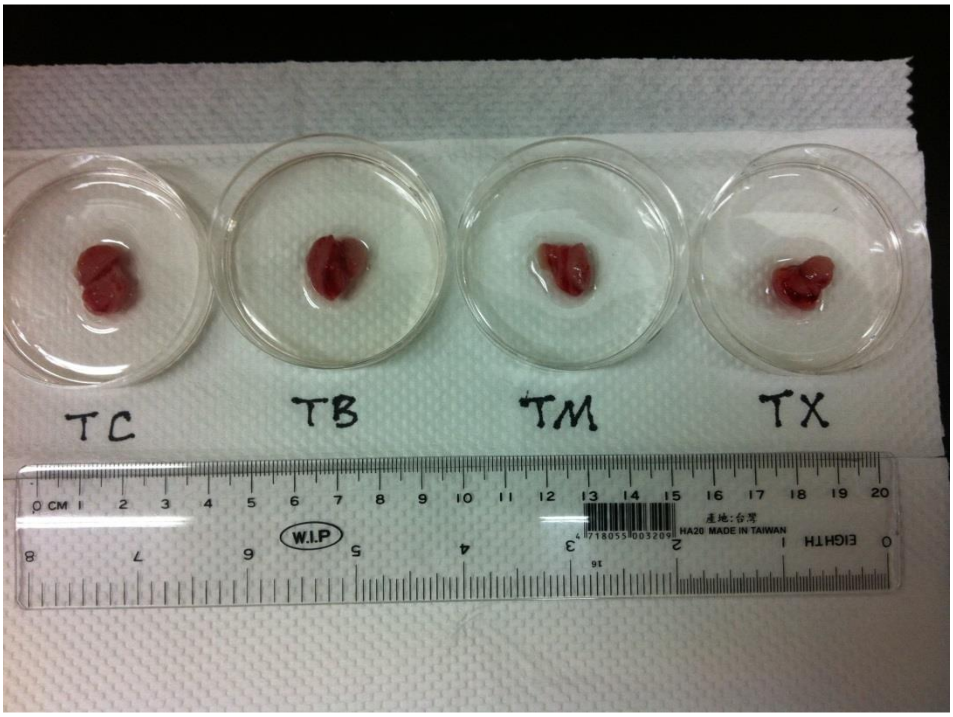

Supplement Data 2. HPLC analysis of *Genoderma lucidum* polysaccharides

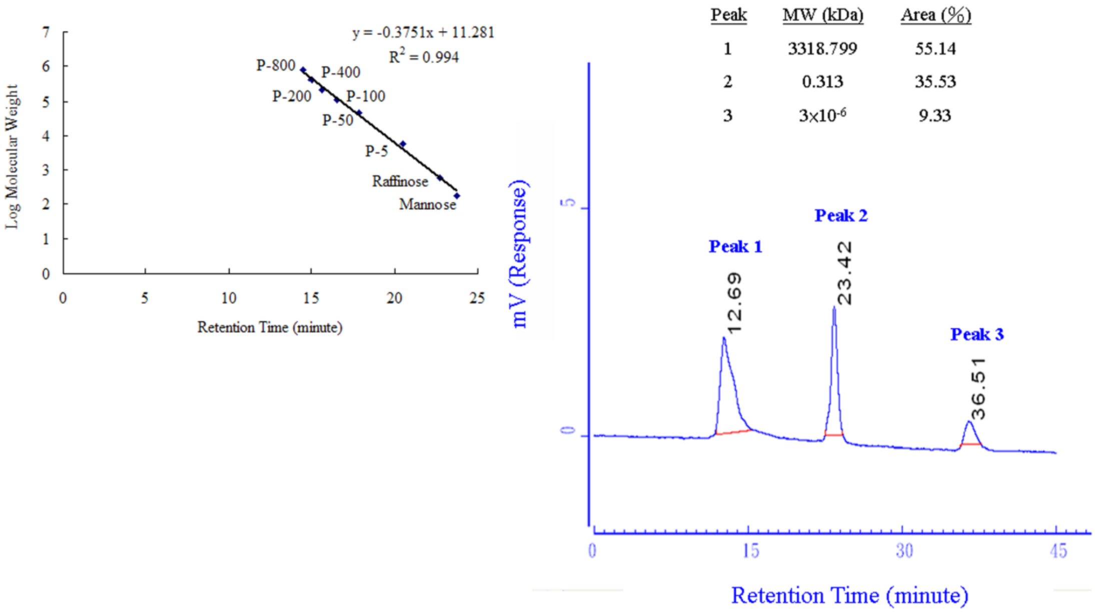

Supplement Data 3. HPLC analysis of *Ganoderma lucidum* polysaccharides

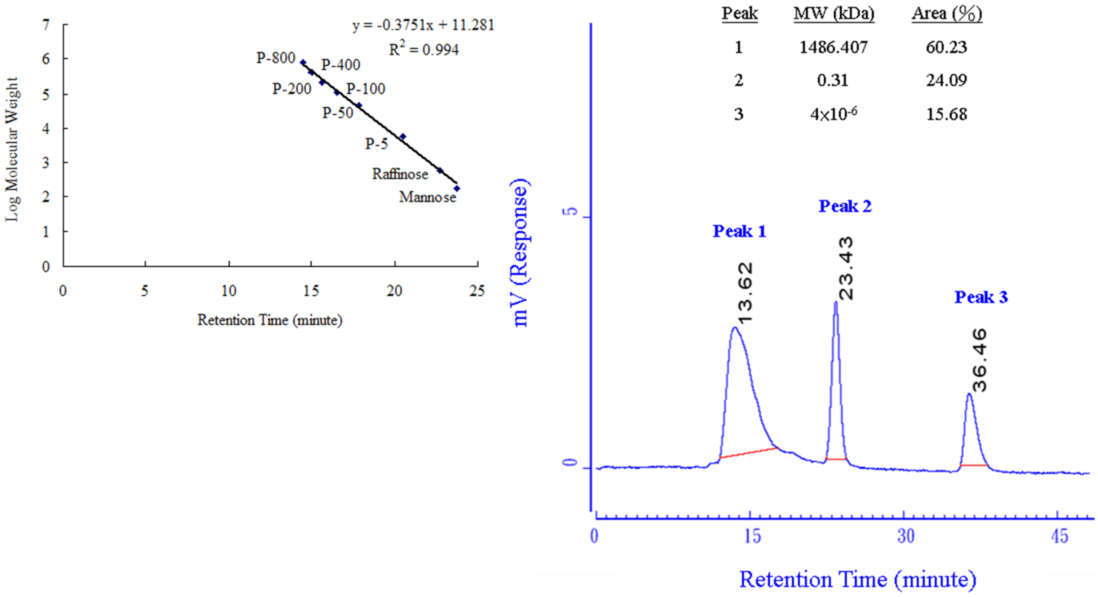

Supplement Data 4. The Flow cytometry analysis

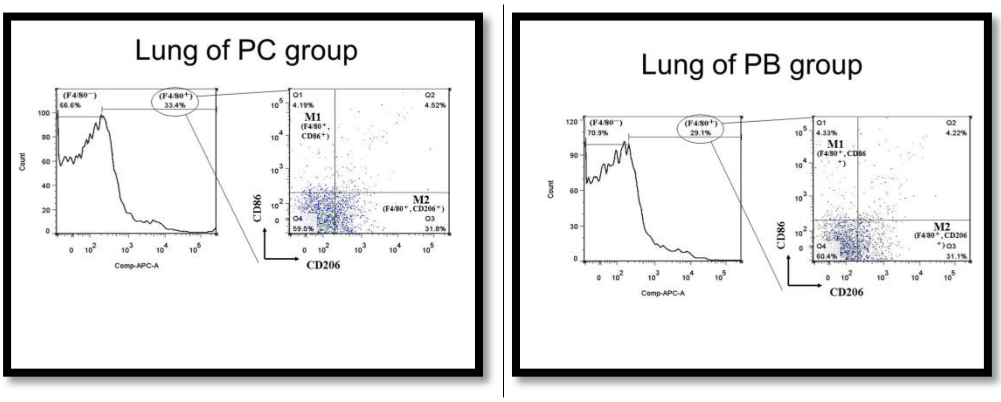

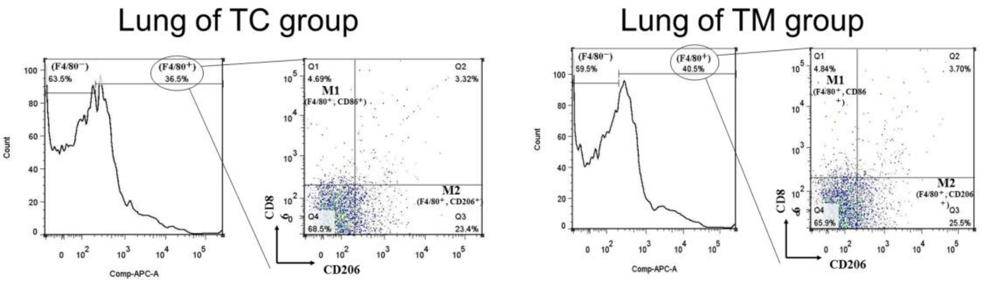
Lung of TC group
Lung of TM group
(F4/80⁻) 63.5%
(F4/80⁺) 36.5%
(F4/80⁻) 59.5%
(F4/80⁺) 40.5%
Count
Comp-APC-A
CD8
CD206
M1
M2
Q1 4.69%
Q2 3.32%
Q3 23.4%
Q4 68.5%
Q1 4.84%
Q2 3.70%
Q3 25.5%
Q4 65.9%

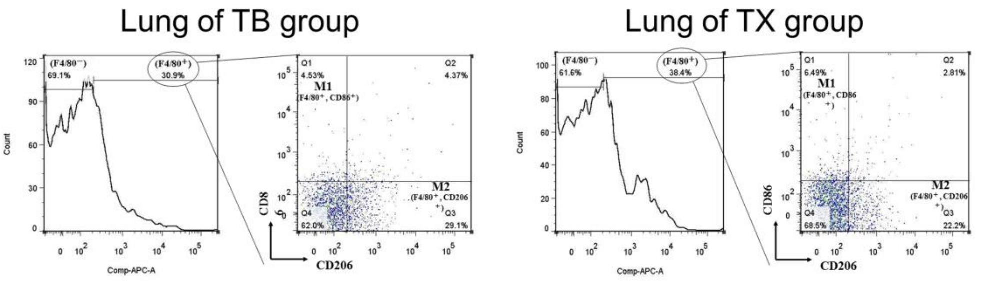
Lung of TB group
Lung of TX group
(F4/80⁻) 69.1%
(F4/80⁺) 30.9%
(F4/80⁻) 61.6%
(F4/80⁺) 38.4%
Count
Comp-APC-A
CD8
CD86
CD206
M1
M2
Q1 4.53%
Q2 4.37%
Q3 29.1%
Q4 62.0%
Q1 6.49%
Q2 2.81%
Q3 22.2%
Q4 68.5%

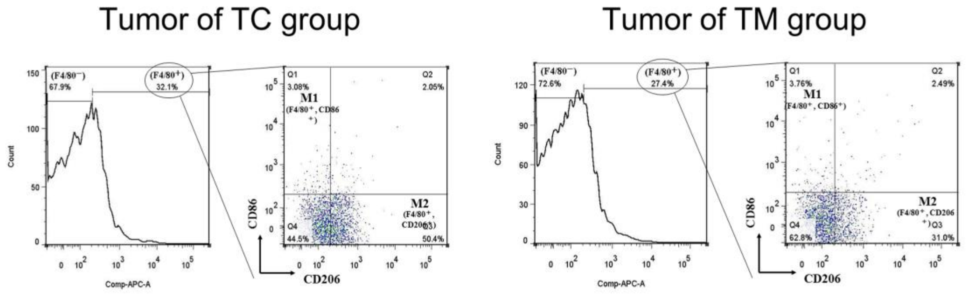
Tumor of TC group
Tumor of TM group
(F4/80⁻) 67.9%
(F4/80⁺) 32.1%
(F4/80⁻) 72.6%
(F4/80⁺) 27.4%
Count
Comp-APC-A
CD86
CD206
M1
M2
Q1 3.08%
Q2 2.05%
50.4%
Q4 44.5%
Q1 3.76%
Q2 2.49%
Q3 31.0%
Q4 62.8%

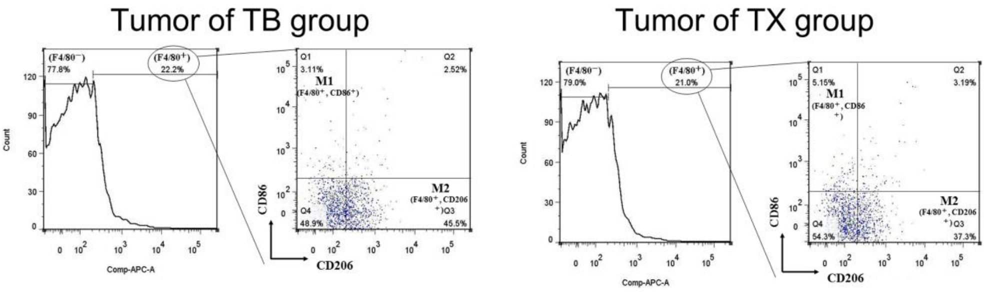
Tumor of TB group
Tumor of TX group
(F4/80⁻) 77.8%
(F4/80⁺) 22.2%
(F4/80⁻) 79.0%
(F4/80⁺) 21.0%
Count
Comp-APC-A
CD86
CD206
M1
M2
Q1 3.11%
Q2 2.52%
Q3 45.5%
Q4 48.9%
Q1 5.15%
Q2 3.19%
Q3 37.3%
Q4 54.3%
